# Potential role of serum hypoxia-inducible factor 1alpha as a biomarker of delayed cerebral ischemia and poor clinical outcome after human aneurysmal subarachnoid hemorrhage: A prospective, longitudinal, multicenter, and observational study

**DOI:** 10.3389/fneur.2022.1072351

**Published:** 2022-12-08

**Authors:** Ye-Yan Cai, Yao-Kun Zhuang, Wen-Jian Wang, Feng Jiang, Jie-Miao Hu, Xiao-Le Zhang, Li-Xin Zhang, Xiao-Hui Lou

**Affiliations:** ^1^Department of Neurosurgery, The Third Affiliated Hospital of Wenzhou Medical University, Ruian People's Hospital, Ruian, China; ^2^Department of Neurosurgery, Ningbo Branch, Ren Ji Hospital, Shanghai Jiao Tong University School of Medicine, Ningbo, China; ^3^Department of Neurosurgery, Hangzhou Ninth People's Hospital, Hangzhou, China

**Keywords:** aneurysm, subarachnoid hemorrhage, hypoxia-inducible factor 1alpha, delayed cerebral ischemia, prognosis, severity

## Abstract

**Objective:**

Hypoxia-inducible factor 1alpha (HIF-1α) functions as a crucial transcriptional mediator in hypoxic and ischemic brain response. We endeavored to assess the prognostic significance of serum HIF-1α in human aneurysmal subarachnoid hemorrhage (aSAH).

**Methods:**

In this prospective, longitudinal, multicenter, and observational study of 257 patients with aSAH and 100 healthy controls, serum HIF-1α levels were quantified. Univariate analyses, followed by multivariate analyses, were performed to discern the relationship between serum HIF-1α levels and severity and delayed cerebral ischemia (DCI) plus poststroke 6-month poor outcome [extended Glasgow outcome scale (GOSE) scores of 1–4]. Predictive efficiency was determined under the receiver operating characteristic (ROC) curve.

**Results:**

There were significantly increased serum HIF-lα levels after aSAH, in comparison to controls (median, 288.0 vs. 102.6 pg/ml; *P* < 0.001). Serum HIF-lα levels were independently correlated with Hunt–Hess scores [β, 78.376; 95% confidence interval (CI): 56.446–100.305; *P* = 0.001] and modified Fisher scores (β, 52.037; 95% CI: 23.461–80.614; *P* = 0.002). Serum HIF-lα levels displayed significant efficiency for discriminating DCI risk [area under ROC curve (AUC), 0.751; 95% CI: 0.687–0.815; *P* < 0.001] and poor outcome (AUC, 0.791; 95% CI: 0.736–0.846; *P* < 0.001). Using the Youden method, serum HIF-1α levels >229.3 pg/ml predicted the development of DCI with 92.3% sensitivity and 48.4% specificity and serum HIF-1α levels >384.0 pg/ml differentiated the risk of a poor prognosis with 71.4% sensitivity and 81.1% specificity. Serum HIF-1α levels >229.3 pg/ml were independently predictive of DCI [odds ratio (OR), 3.061; 95% CI: 1.045–8.965; *P* = 0.041] and serum HIF-1α levels >384.0 pg/ml were independently associated with a poor outcome (OR, 2.907; 95% CI: 1.403–6.024; *P* = 0.004). The DCI predictive ability of their combination was significantly superior to those of Hunt–Hess scores (AUC, 0.800; 95% CI: 0.745–0.855; *P* = 0.039) and modified Fisher scores (AUC, 0.784; 95% CI: 0.726–0.843; *P* = 0.004). The prognostic predictive ability of their combination substantially exceeded those of Hunt–Hess scores (AUC, 0.839; 95% CI: 0.791–0.886; *P* < 0.001) and modified Fisher scores (AUC, 0.844; 95% CI: 0.799–0.890; *P* < 0.001).

**Conclusion:**

Elevated serum HIF-lα levels after aSAH, in independent correlation with stroke severity, were independently associated with DCI and 6-month poor outcome, substantializing serum HIF-lα as a potential prognostic biomarker of aSAH.

## Introduction

Aneurysmal subarachnoid hemorrhage (aSAH) affects six to nine people per 100,000 per year, accounts for 5% of the hemorrhagic stroke, and is characterized by a high rate of morbidity and mortality (1). In addition to diagnosis and treatment, prognosis prediction is a very important aspect of aSAH therapy (2). Hunt–Hess scale and modified Fisher scale are often used to assess illness severity and predict clinical outcomes of aSAH (3). Extended Glasgow outcome scale (GOSE) is conventionally regarded as a prognostic parameter in some brain injury diseases, including aSAH (4). Delayed cerebral ischemia (DCI) is a usually observed adverse event after aSAH, which is associated with an increased risk of a poor outcome for patients with aSAH (5). Its occurrence may involve large artery vasospasm and/or microcirculatory disturbances by micro vasospasm, microthrombosis, dysfunction of venous outflow, and compression of microvasculature by vasogenic or cytotoxic tissue edema (5). In addition to DCI, early brain injury, which is identified as another pathophysiological event of secondary brain injury after aSAH, involves some key pathophysiological mechanisms, including brain ischemia and hypoxia, thereby inducing neuronal death and finally causing neurologic dysfunction (6). Because peripheral blood is a kind of easily obtained biofluid, biochemical markers in peripheral blood have attracted extensive interest as prognostic parameters of aSAH during recent decades (7–9).

Hypoxia-inducible factor-1 (HIF-1), an oxygen-sensitive transcriptional activator, regulates the expression of a series of genes that facilitate the adaptation to low oxygen tension (hypoxia) in cells and tissues (10). HIF-1alpha (1α) is a subunit (11) whose expressions by neurons and astrocytes were markedly upregulated following experimental SAH, ischemic stroke, or intracerebral hemorrhage (12–15). Up to now, there are inconsistent data available regarding the actual effect of HIF-1α in acute brain injury, whether protective or detrimental (15–21). In a study of 40 patients with acute ischemic stroke, elevated serum HIF-1α levels were closely related to cerebral infarction size (22). Alternatively, in two cohort studies of almost 100 patients with spontaneous intracerebral hemorrhage or severe traumatic brain injury, there was a significant increase in serum HIF-1α levels, which, in intimate correlation with illness severity, independently predicted poor prognosis (Glasgow outcome scale scores of 1–3) at 90 days after injury (23, 24). However, to the best of our knowledge, HIF-1α levels have not been measured in peripheral blood of humans with aSAH. Herein, we undertook a multicenter study to further ascertain whether serum HIF-1α could be associated with the illness severity and long-term clinical outcome of humans with aSAH.

## Methods

### Study design, subject selection, and ethics approval

This prospective longitudinal observational study was performed from April 2018 to April 2021 at three hospitals, including Ruian People's Hospital, the Third Affiliated Hospital of Wenzhou Medical University; Ningbo Branch, Renji Hospital, Shanghai Jiao Tong University School of Medicine; and Hangzhou Ninth People's Hospital. We consecutively enrolled patients with nontraumatic SAH, who were diagnosed *via* head computed tomography (CT) scan. Those patients were entered into the study if they followed the inclusion criteria as listed below: (1) first-onset spontaneous SAH, (2) adults (age equal to or more than 18 years), (3) SAH resulting from rupture of a single intracranial aneurysm, (4) hospitalization of 24 h following SAH, and (5) aneurysmal treatment of 48 h after hospital admission. Some patients should be excluded in agreement with the following exclusion criteria: (1) occurrence of aneurysm rebleeding, (2) suspected pseudoaneurysm, (3) previous neurologic diseases, such as stroke, severe head trauma, myasthenia gravis, and intracranial tumors, and (4) other specific diseases or conditions, such as pregnancies, malignancies, chronic heart, liver, kidney, and lung diseases, and other coexisting acute severe illnesses, including acute myocardial infarction and acute lung injury. From April 2020 to April 2021, a group of healthy volunteers was recruited as controls at the preceding three hospitals. Controls were free of other diseases, such as hypertension, diabetes mellitus, and chronic heart disease. Some routine tests, including blood glucose, hemoglobin, and sodium and potassium levels, as well as blood leucocyte, platelet, and neutrophil counts, were normal in controls. The protocol of the current study was established, and the study was carried out in compliance with the Declaration of Helsinki. Also, approval for the protocol of this study was acquired from the Ethical Committees of the aforementioned three hospitals. Patients' relatives or controls themselves signed informed consent for participating in this study.

### Data collection and clinical and outcome assessment

Upon entry into the emergency center, we inquired about demographics (age and gender), hospital admission time, vascular risk factors, including two adverse life habits (cigarette smoking and alcohol drinking) and three chronic illnesses (hypertension, diabetes mellitus, and hyperlipidemia), and medication history (use of statins, anticoagulation drugs, and antiplatelet drugs). Hunt–Hess scale was considered as a clinical severity indicator and the modified Fisher scale was regarded as a radiological severity parameter. Aneurysm-related radiological characteristics were obtained using CT angiography or digital subtraction angiography (DSA). Specifically, the position was divided into posterior and anterior circulation, the shape was classified into cystic and others, and the size was assigned into diameters < 10 mm and ≥10 mm. Therapeutic modalities for aneurysms included neurosurgical clipping and endovascular interventional embolization. Two acute adverse events were acute hydrocephalus and intraventricular hemorrhage, which were confirmed *via* head CT scans. External ventricular drainage was a surgical choice for such adverse events. During in-hospital treatments, DCI was determined in compliance with the previously established criteria as follows: (1) clinical deterioration (namely, a new focal deficit, decrease in the level of consciousness, or both) and/or (2) a new infarct on the head CT scan that was invisible at admission or immediately postoperatively, and cannot be attributed to other causes by means of clinical assessment, imaging of the brain, and appropriate laboratory studies (25). Extended Glasgow outcome scale (GOSE), ranging from 1 to 8, was recorded for assessing functional outcomes of patients with aSAH at 6 months post-injury. GOSE scores of 1–4 were referred to as a poor outcome (26).

### Measurement of serum HIF-1α levels

Blood samples were promptly obtained upon patients' entry into the emergency department and those of controls were acquired when entering into the study. After blood was centrifugated, serum was aliquoted, and then stored at −80°C until measurements. A commercially available human HIF-1α enzyme-linked immunosorbent assay kit (RapidBio Lab, California, USA) was used for the determination of serum HIF-1α based on the manufacturer's instructions. The determinations of serum HIF-1a were in batches done every 3 months. Serum HIF-1α levels were in duplicate detected by the same technician who was inaccessible to the clinical data, and accordingly, the mean value of two measurements was utilized for further statistical analysis.

### Statistical analysis

Statistical analysis was performed using three statistical software, namely, R software (version 3.5.1; https://www.r-project.org), SPSS 19.0 (SPSS Inc., Chicago, IL, USA), and MedCalc 9.6.4.0 (MedCalc Software, Mariakerke, Belgium). Graphs were plotted using GraphPad Prism software version 6.0 for Windows (GraphPad Software, San Diego, California, USA). Qualitative variables, which were shown as frequencies (proportions), were compared between two groups using the Chi-square test or Fisher exact test where appropriate. Normally distributed quantitative variables, which were reported as means (standard deviations, SD), were compared between two groups using an independent t-test. Non-normally distributed quantitative variables, which were presented as medians (percentiles 25th−75th), were compared between two groups using the Mann–Whitney *U*-test. The Kruskal–Wallis test was performed to be a comparative of serum HIF-1α levels among multiple groups with different GOSE scores, Hunt–Hess scores, or modified Fisher scores. The Spearman test, followed by the multivariate linear regression analysis, was used to ascertain the independent relationships between serum HIF-1α levels and hemorrhagic severity, which were indicated by Hunt–Hess scores and modified Fisher scores. The binary logistic regression models were built to discern predictors, which were in an independent relation with DCI and 6-month poor outcome. The area under the receiver operating characteristic (ROC) curve (AUC) was calculated to reflect the predictive efficiency. Using the Youden method, an optimal cutoff value of serum HIF-1α levels was chosen, thereby generating the corresponding sensitivities and specificities. Using the *Z*-test, AUCs were compared. Two-tailed *P*-values of < 0.05 indicated statistically significant differences.

## Results

### Participants' characteristics

**Figure 1:**
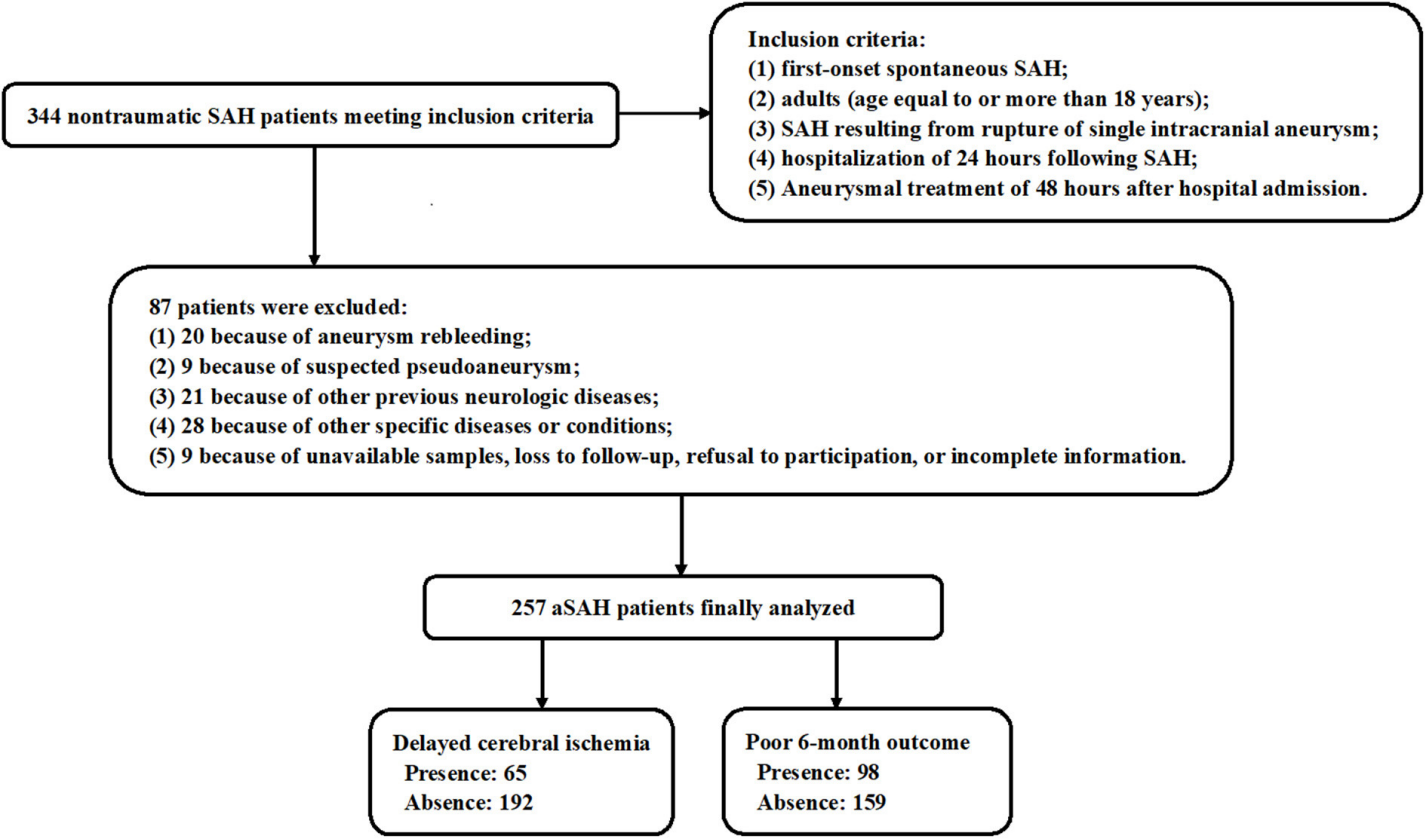
Flow chart for screening patients with aneurysmal subarachnoid hemorrhage. A total of 344 patients with spontaneous aneurysmal subarachnoid hemorrhage obtained an initial assessment according to the inclusion criteria, then 87 patients were removed from this study based on the exclusion criteria, and finally, 257 patients were retained for further analysis. aSAH, aneurysmal subarachnoid hemorrhage.

During the study period, 344 nontraumatic patients with aSAH fitted the preset inclusion criteria, and then, a total of 257 patients with aSAH were retained for final analysis after 87 patients were excluded because of the reasons presented in Figure 1. Also, 100 healthy individuals constituted controls. Some baseline characteristics of controls were presented in Table 1. Age, as well as smoker, drinker, and gender percentages, did not significantly differ between patients and controls (all *P* > 0.05; Table 1).

**Table 1:** Differences in baseline characteristics between healthy controls and patients with aneurysmal subarachnoid hemorrhage.

	**Patients**	**Controls**	***P-*value**
Gender (male/female)	113/144	44/56	0.996
Age (years)	51.1 ± 10.6	50.6 ± 13.1	0.214
Cigarette smoking	83 (32.3%)	31 (31.0%)	0.814
Alcohol drinking	66 (25.7%)	28 (28.0%)	0.655

Variables were reported as count (proportion) or mean ± standard deviation as appropriate. Statistical methods included the student t-test and χ^2^ test.

Among 257 patients with aSAH, there were 113 males and 144 females, and their age ranged from 30 years to 73 years (mean, 51.1 years; SD, 10.6 years). There were 83 cigarette smokers and 66 alcohol drinkers. A total of 53, 23, and 38 patients were inflicted with hypertension, diabetes mellitus, and hyperlipidemia, respectively. In aggregate, 21, 13, and 12 patients previously orally took statins, anticoagulation drugs, and antiplatelet drugs, respectively. Regarding aneurysm-related radiological characteristics, there were 153 aneurysms of < 10 mm in diameter, 207 aneurysms located in the anterior circulation, and 215 cystic aneurysms. Patients were admitted from 0.5 to 24.0 h (median, 10.3 h; lower–upper quartiles, 4.9–14.5 h) after injury. Blood was collected from 1.0 to 26.0 h (median, 11.7 h; percentiles 25th−75th, 6.6–16.3 h) after stroke. Hunt–Hess scores ranged from 1 to 5 (median, 3; lower–upper quartiles, 2–4). Modified Fisher scores ranged from 1 to 4 (median, 2; lower–upper quartiles, 2–3). Hunt–Hess scores of 1, 2, 3, 4, and 5 were found in 41, 59, 86, 57, and 14 patients, respectively. Modified Fisher scores of 1, 2, 3, and 4 were revealed in 46, 115, 72, and 24 patients, respectively. In total, 161 patients underwent an initial endovascular intervention for securing aneurysms and surgical clipping was done in other remainders. Totally, 32 patients were complicated with acute hydrocephalus, 26 patients suffered from intraventricular bleeding, and 33 patients accepted external drainage for the removal of intraventricular bleeding.

### Serum HIF-1α levels between patients with aSAH and controls and its correlation with hemorrhagic severity

**Figure 2:**
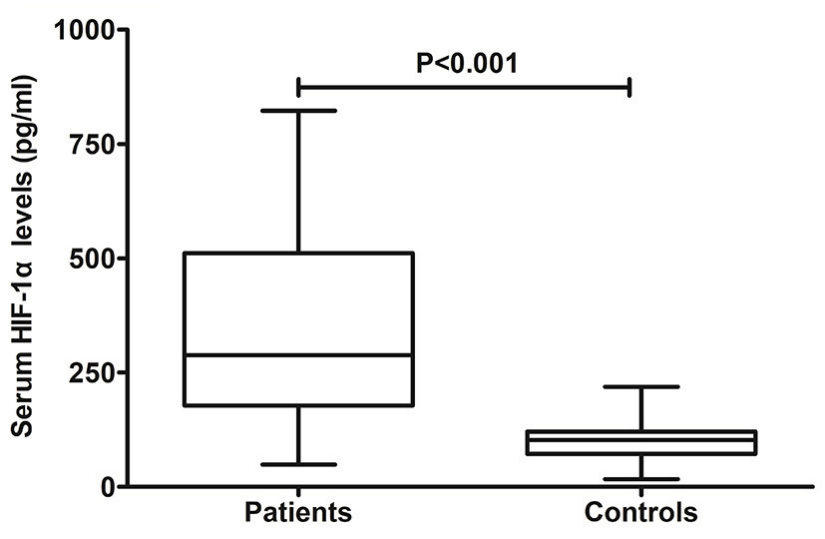
Change of serum hypoxia-inducible factor 1alpha levels after aneurysmal subarachnoid hemorrhage. Serum hypoxia-inducible factor 1alpha levels were significantly elevated in comparison to healthy controls (*P* < 0.001). HIF-1α denotes hypoxia-inducible factor 1alpha.

**Figure 3:**
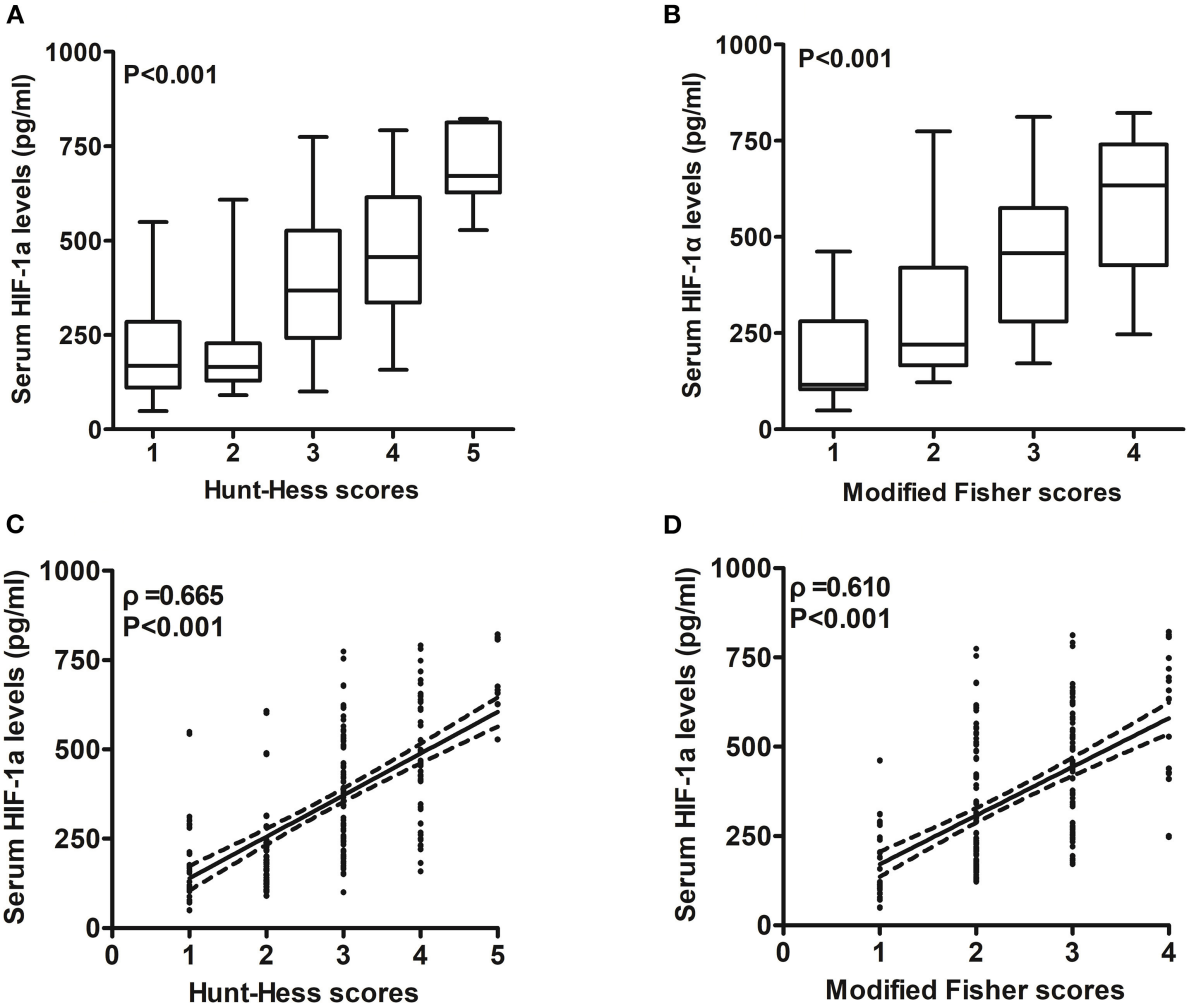
Relationship between serum hypoxia-inducible factor 1alpha levels and illness severity after aneurysmal subarachnoid hemorrhage. **(A)** Serum hypoxia-inducible factor 1alpha levels among patients with different Hunt–Hess scores. Serum hypoxia-inducible factor 1alpha levels were substantially lowest in patients with Hunt–Hess score of 1, followed by Hunt–Hess scores of 2, 3, and 4, and were markedly highest in those with Hunt–Hess score of 5 (*P* < 0.001). **(B)** Serum hypoxia-inducible factor 1alpha levels among patients with different modified Fisher scores. Serum hypoxia-inducible factor 1alpha levels were substantially lowest in patients with modified Fisher scores of 1, followed by modified Fisher scores of 2 and 3, and were markedly highest in those with modified Fisher scores of 4 (*P* < 0.001). **(C)** Relation of serum hypoxia-inducible factor 1alpha levels to Hunt–Hess scores. Serum hypoxia-inducible factor 1alpha levels were substantially correlated with Hunt–Hess scores (*P* < 0.001). **(D)** Relation of serum hypoxia-inducible factor 1alpha levels to modified Fisher scores. Serum hypoxia-inducible factor 1alpha levels were substantially correlated with modified Fisher scores (*P* < 0.001). HIF-1α means hypoxia-inducible factor 1alpha.

In Figure 2, patients with aSAH displayed substantially higher serum HIF-1α levels than controls (*P* < 0.001). In Table 2, serum HIF-1α levels were tightly correlated with other variables, namely, Hunt–Hess scores, modified Fisher scores, acute hydrocephalus, intraventricular hemorrhage, external ventricular drainage, and blood glucose levels (all *P* < 0.05). The significantly correlated variables were forced into the multivariate linear regression model, and afterward, serum HIF-1α levels were independently correlated with Hunt–Hess scores [β, 78.376; 95% confidence interval (CI): 56.446–100.305; *P* = 0.001] and modified Fisher scores (β, 52.037; 95% CI: 23.461–80.614; *P* = 0.002). Just as graphed in Figure 3, serum HIF-1α levels were highly correlated with Hunt–Hess scores and modified Fisher scores, whether they were identified as categorical or continuous variables (all *P* < 0.001).

**Table 2:** Factors related to serum hypoxia-inducible factor 1alpha levels following aneurysmal subarachnoid hemorrhage.

	**ρ**	***P-*value**
Male	−0.081	0.194
Age (years)	0.089	0.155
Cigarette smoking	0.012	0.843
Alcohol drinking	0.072	0.249
Hypertension	0.020	0.752
Diabetes mellitus	0.109	0.082
Hyperlipidemia	0.081	0.194
Previous use of statins	0.000	0.994
Previous use of anticoagulation drugs	0.012	0.853
Previous use of antiplatelet drugs	−0.057	0.363
Hunt-Hess scores	0.665	< 0.001
Modified Fisher scores	0.610	< 0.001
Aneurysms located at anterior circulation	−0.091	0.148
Cystic aneurysms	−0.058	0.351
Aneurysms with diameter of < 10 mm	−0.064	0.304
Surgical clipping for securing aneurysms	−0.049	0.437
Acute hydrocephalus	0.224	< 0.001
Intraventricular bleeding	0.191	0.002
External ventricular drain	0.194	0.002
Admission time after stroke (h)	0.098	0.117
Blood-collection time after stroke (h)	0.110	0.078
Blood glucose levels (mmol/L)	0.236	< 0.001
Blood leukocyte count ( × 10^9^/L)	0.022	0.725

Bivariate correlations were assessed by Spearman test.

### Serum HIF-1α levels and DCI

**Figure 4:**
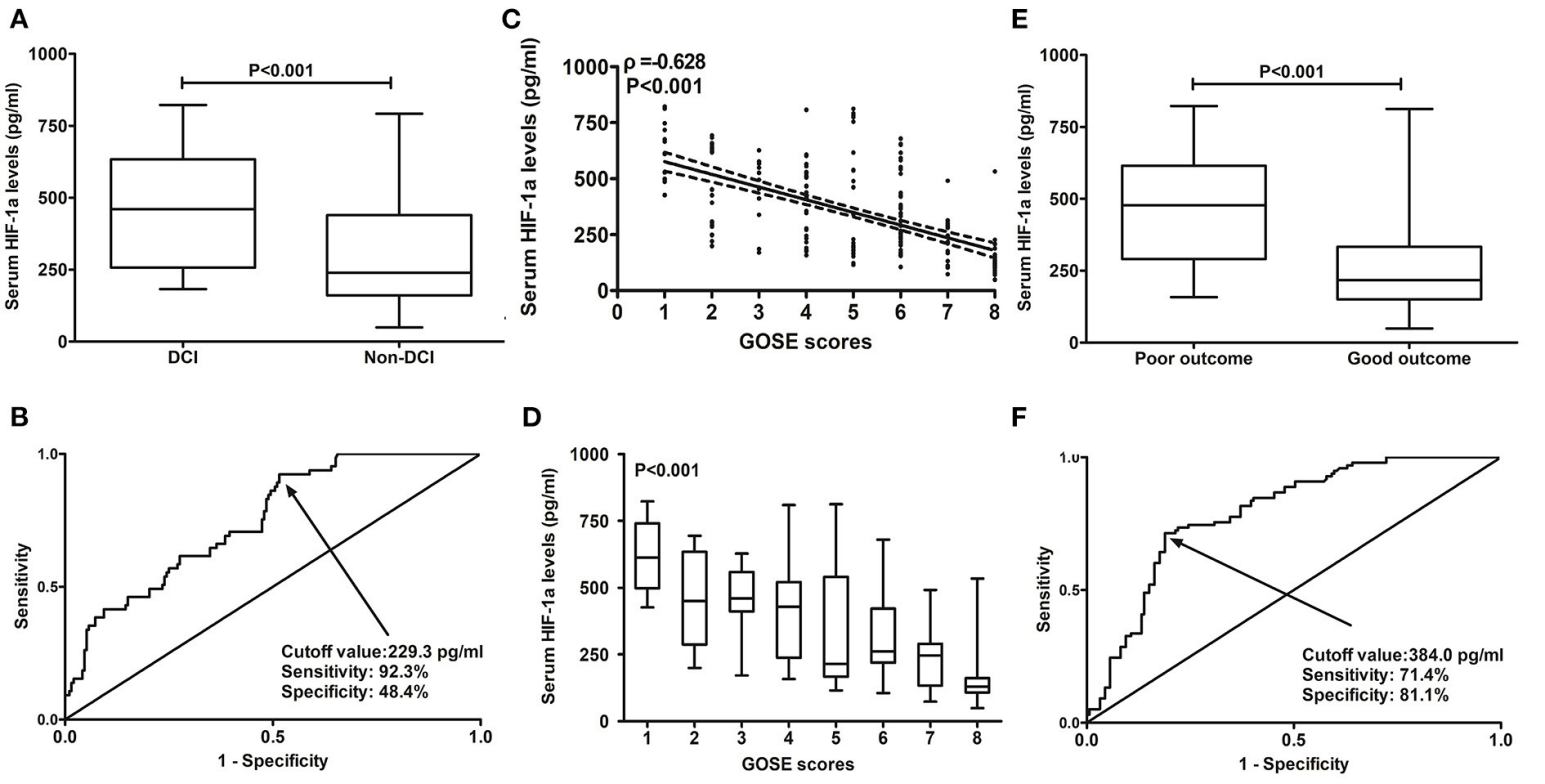
Predictive ability of serum hypoxia-inducible factor 1alpha levels for patients at risk of delayed cerebral ischemia and 6-month poor outcome after aneurysmal subarachnoid hemorrhage. **(A)** Serum hypoxia-inducible factor 1alpha levels between patients with delayed cerebral ischemia and those not presenting with delayed cerebral ischemia. Serum hypoxia-inducible factor 1 alpha levels were profoundly higher in patients with delayed cerebral ischemia than in other remainders (*P* < 0.001). **(B)** Discriminatory power of serum hypoxia-inducible factor 1alpha levels for risk of delayed cerebral ischemia. Serum hypoxia-inducible factor 1alpha levels substantially distinguished the development of delayed cerebral ischemia (*P* < 0.001). Using the Youden method, an optimal cutoff value was chosen, which produced the corresponding sensitivity and specificity values. **(C)** Relation of serum hypoxia-inducible factor 1alpha levels to extended Glasgow outcome scale scores. Serum hypoxia-inducible factor 1alpha levels were substantially correlated with extended Glasgow outcome scale scores (*P* < 0.001). **(D)** Serum hypoxia-inducible factor 1alpha levels among patients with different extended Glasgow outcome scale scores. Serum hypoxia-inducible factor 1alpha levels were substantially highest in patients with extended Glasgow outcome scale score of 1, followed by extended Glasgow outcome scale scores of 2–7, and were markedly lowest in those with extended Glasgow outcome scale score of 8 (*P* < 0.001). **(E)** Serum hypoxia-inducible factor 1alpha levels between patients with poor outcomes and those with good outcomes. Serum hypoxia-inducible factor 1 alpha levels were profoundly higher in patients with poor outcomes than in those with good outcomes (*P* < 0.001). **(F)** Distinguishable value of serum hypoxia-inducible factor 1alpha levels for poor outcomes. Serum hypoxia-inducible factor 1alpha levels pronouncedly discriminated poor outcomes (*P* < 0.001). Using the Youden method, an optimal cutoff value was selected, which yielded the corresponding sensitivity and specificity values. HIF-1α, hypoxia-inducible factor 1alpha; GOSE, extended Glasgow outcome scale; DCI, delayed cerebral ischemia.

A total of 65 patients (25.3%) with DCI were found in this cohort of patients with aSAH. Serum HIF-1α levels were significantly higher in patients with DCI than in those without DCI (*P* < 0.001; Figure 4A). Under the ROC curve, serum HIF-1α levels distinguished patients at significant risk of DCI (AUC, 0.751; 95% CI: 0.687–0.815; *P* < 0.001). Using the Youden method, serum HIF-1α levels of >229.3 pg/ml predicted the development of DCI with medium–high sensitivity and specificity values (Figure 4B).

In Table 3, as compared to patients not presenting with DCI, those suffering from DCI exhibited substantially elevated Hunt–Hess scores, modified Fisher scores, and blood glucose (all *P* < 0.05), as well as had significantly higher percentages of serum HIF-1α levels of >229.3 pg/ml, acute hydrocephalus, intraventricular hemorrhage, and external ventricular drainage (all *P* < 0.05). Thereafter, the aforementioned variables were forced into the binary logistic regression model, and subsequently, it was shown that serum HIF-1α levels of >229.3 pg/ml [odds ratio (OR), 3.061; 95% CI: 1.045–8.965; *P* = 0.041], Hunt–Hess scores (OR, 1.972; 95% CI: 1.233–3.155; *P* = 0.005), and modified Fisher scores (OR, 1.977; 95% CI: 1.167–3.351; *P* = 0.011) were the three independent predictors of DCI after aSAH.

**Table 3:** Factors in relation to delayed cerebral ischemia following aneurysmal subarachnoid hemorrhage.

	**DCI**	**Non-DCI**	***P-*value**
Male	30 (46.2%)	83 (43.2%)	0.681
Age (years)	52.0 ± 10.6	50.8 ± 10.6	0.455
Cigarette smoking	22 (33.9%)	61 (31.8%)	0.757
Alcohol drinking	18 (27.7%)	48 (25.0%)	0.668
Hypertension	14 (21.5%)	39 (20.3%)	0.833
Diabetes mellitus	9 (13.9%)	14 (7.3%)	0.110
Hyperlipidemia	10 (15.4%)	28 (14.6%)	0.875
Previous use of statins	5 (7.7%)	16 (8.3%)	0.870
Previous use of anticoagulation drugs	3 (4.6%)	10 (5.2%)	0.850
Previous use of antiplatelet drugs	5 (7.7%)	7 (3.7%)	0.186
Hunt-Hess scores	4 (3–4)	2.5 (2–3)	< 0.001
Modified Fisher scores	3 (2–3)	2 (2–2.5)	< 0.001
Aneurysms located at anterior circulation	52 (80.0%)	155 (80.7%)	0.898
Cystic aneurysms	51 (78.5%)	164 (85.4%)	0.190
Aneurysms with diameter of < 10 mm	36 (55.4%)	117 (60.9%)	0.430
Surgical clipping for securing aneurysms	19 (29.2%)	77 (40.1%)	0.117
Acute hydrocephalus	17 (26.2%)	15 (7.8%)	< 0.001
Intraventricular bleeding	11 (16.9%)	15 (7.8%)	0.035
External ventricular drain	15 (23.1%)	18 (9.4%)	0.004
Admission time after stroke (h)	9.5 (4.9–13.7)	10.8 (5.0–14.5)	0.992
Blood-collection time (h)	11.2 (6.5–16.0)	12.1 (6.6–16.5)	0.955
Blood glucose levels (mmol/L)	12.3 (8.6–16.7)	10.7 (8.5–12.9)	0.035
Blood leukocyte count ( × 10^9^/L)	8.1 (6.3–10.9)	7.6 (6.1–10.6)	0.568
Serum HIF-1α levels >229.3 pg/ml	60 (92.3%)	99 (51.6%)	< 0.001

Variables were reported as count (proportion), mean ± standard deviation, or median (percentiles 25th−75th) where appropriate. Statistical methods included the student t-test, Mann–Whitney test, Fisher's exact test, and χ^2^ test. DCI denotes delayed cerebral ischemia. HIF-1α means hypoxia-inducible factor 1alpha.

**Figure 5:**
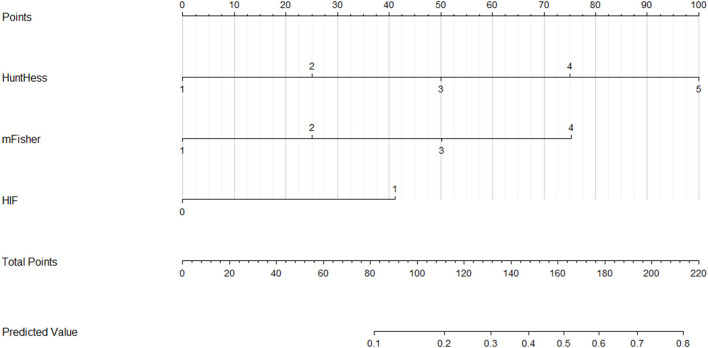
Nomogram for predicting delayed cerebral ischemia among patients with aneurysmal subarachnoid hemorrhage. Serum hypoxia-inducible factor 1alpha levels combined with Hunt–Hess scores and modified Fisher scores significantly discriminated against patients at risk of delayed cerebral ischemia. For HIF, “1” indicates serum hypoxia-inducible factor 1alpha levels >229.3 pg/ml and “0” means serum hypoxia-inducible factor 1alpha levels <229.3 pg/ml. HuntHess, Hunt–Hess scores; mFisher, modified Fisher scores; HIF, hypoxia-inducible factor 1alpha.

**Figure 6:**
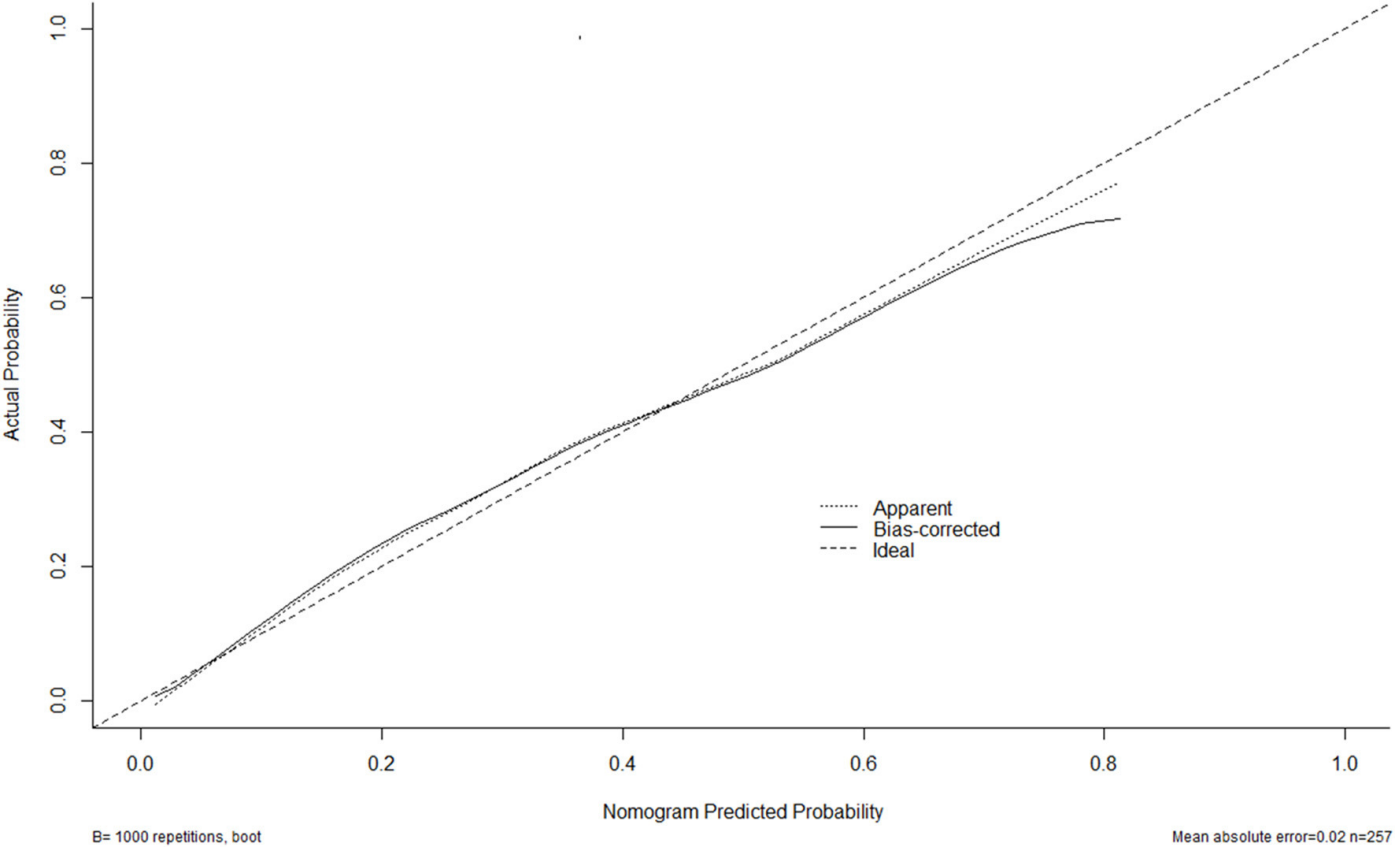
Calibration curves of the nomogram for predicting delayed cerebral ischemia among patients with aneurysmal subarachnoid hemorrhage. The *x*-axis denotes the predicted delayed cerebral ischemia. The *y*-axis denotes the actual delayed cerebral ischemia. The diagonal dotted line means a perfect prediction using an ideal model. The solid line represents the performance of the nomogram, of which the closer fit to the diagonal dotted line indicates the better prediction of the nomogram.

**Figure 7:**
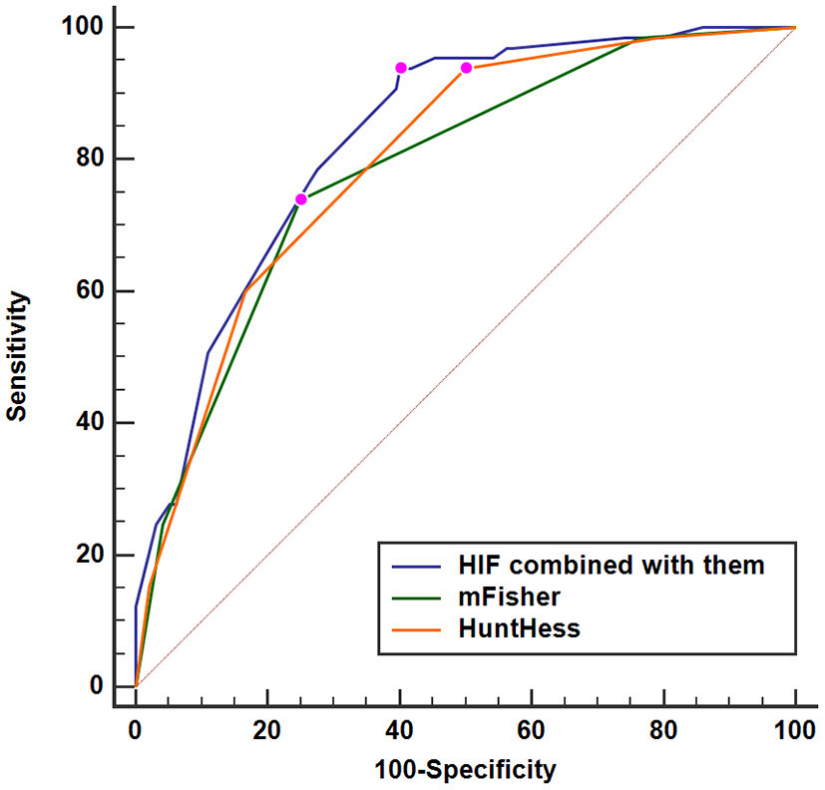
Predictive ability with respect to serum hypoxia-inducible factor 1alpha levels combined with Hunt–Hess scores and modified Fisher scores for the risk of delayed cerebral ischemia. Serum HIF-1α levels combined with Hunt–Hess scores and modified Fisher scores had significantly increased predictive ability for the risk of delayed cerebral ischemia, as compared to Hunt–Hess scores and modified Fisher scores alone (*P* < 0.05). HuntHess, Hunt–Hess scores; mFisher, modified Fisher scores; HIF, hypoxia-inducible factor 1alpha.

A nomogram was drawn to obtain a more comprehensive view of the relationship between DCI risk and other independent predictors, namely, Hunt–Hess scores, modified Fisher scores, and serum HIF-1α levels of >229.3 pg/ml (Figure 5). The calibration curve of the nomogram for distinguishing DCI risk showed that the mean absolute error was 0.020 (Figure 6). The combined binary logistic regression model was built, which included Hunt–Hess scores, modified Fisher scores, and serum HIF-1α levels of >229.3 pg/ml (using Hosmer and Lemeshow test, *P* = 0.908). Subsequently, serum HIF-1α levels of >229.3 pg/ml combined with Hunt–Hess scores and modified Fisher scores had AUC at 0.832 (95% CI: 0.780–0.884). In Figure 7, the DCI predictive ability of their combination was significantly superior to those of Hunt–Hess scores (AUC, 0.800; 95% CI: 0.745–0.855; *P* = 0.039) and modified Fisher scores (AUC, 0.784; 95% CI: 0.726–0.843; *P* = 0.004).

### Serum HIF-1α levels and 6-month poor prognosis

At 6 months following aSAH, GOSE scores ranged from 1 to 8, with a median value of 5 (lower–upper quartiles, 4–7), and a total of 16, 29, 17, 36, 31, 61, 27, and 40 patients exhibited GOSE scores of 1, 2, 3, 4, 5, 6, 7, and 8, respectively. In Figure 4C, serum HIF-1α levels were substantially and inversely correlated with 6-month GOSE scores (*P* < 0.001), and in Figure 4D, they were statistically significantly highest in patients with the development of GOSE score of 1, followed by GOSE scores of 2, 3, 4, 5, 6, and 7, and were pronouncedly lowest in those presented with GOSE score of 8 (*P* < 0.001).

In aggregate, 98 patients (38.1%) experienced a poor prognosis (GOSE scores 1–4) at 6 months after aSAH. Just as presented in Figure 4E, serum HIF-1α levels were substantially elevated in patients with GOSE scores of 1–4 in comparison with those suffering from GOSE scores of 5–8 (*P* < 0.001). Under the ROC curve, serum HIF-1α levels substantially distinguished patients at risk of a poor prognosis (AUC, 0.791; 95% CI: 0.736–0.846; *P* < 0.001). Using the Youden method, serum HIF-1α levels of >384.0 pg/ml differentiated the risk of a poor prognosis with medium–high sensitivity and specificity values (Figure 4F).

Using univariate analysis, as compared to patients with good outcomes, those with poor outcomes showed substantially increased blood glucose levels, Hunt–Hess scores, and modified Fisher scores (all *P* < 0.05; Table 4), as well as had significantly increased percentages of serum HIF-1α levels of >384.0 pg/ml, previous use of anticoagulation drugs, diabetes mellitus, intraventricular hemorrhage, and hydrocephalus (all *P* < 0.05; Table 4). Afterward, the binary logistic regression model was established, where the preceding significant variables were contained, and it was demonstrated that Hunt–Hess scores, modified Fisher scores, and serum HIF-1α levels of >384.0 pg/ml independently predicted the development of 6-month poor prognosis after aSAH with OR values of 2.086 (95% CI: 1.302–3.342; *P* = 0.002), 4.467 (95% CI: 2.421–8.244; *P* = 0.001), and 2.907 (95% CI: 1.403–6.024; *P* = 0.004), respectively.

**Table 4:** Factors in correlation with 6-month poor prognosis following aneurysmal subarachnoid hemorrhage.

	**Poor prognosis**	**Good prognosis**	***P-*value**
Male	42 (42.9%)	71 (44.7%)	0.778
Age (years)	52.0 ± 11.1	50.6 ± 10.2	0.334
Cigarette smoking	32 (32.7%)	51 (32.1%)	0.923
Alcohol drinking	29 (29.6%)	37 (23.3%)	0.260
Hypertension	24 (24.5%)	29 (18.2%)	0.229
Diabetes mellitus	15 (15.3%)	8 (5.0%)	0.005
Hyperlipidemia	18 (18.4%)	20 (12.6%)	0.204
Previous use of statins	7 (7.1%)	14 (8.8%)	0.637
Previous use of anticoagulation drugs	9 (9.2%)	4 (2.5%)	0.018
Previous use of antiplatelet drugs	6 (6.1%)	6 (3.8%)	0.386
Hunt-Hess scores	4 (3–4)	2 (2–3)	< 0.001
Modified Fisher scores	3 (2–3)	2 (1–2)	< 0.001
Aneurysms located at anterior circulation	80 (81.6%)	127 (79.9%)	0.729
Cystic aneurysms	83 (84.7%)	132 (83.0%)	0.724
Aneurysms with diameter of < 10 mm	53 (54.1%)	100 (62.9%)	0.162
Surgical clipping for securing aneurysms	33 (33.7%)	63 (39.6%)	0.338
Acute hydrocephalus	23 (23.5%)	9 (5.7%)	< 0.001
Intraventricular hemorrhage	15 (15.3%)	11 (6.9%)	0.030
External ventricular drain	17 (17.3%)	16 (10.1%)	0.090
Admission time after stroke (h)	10.1 (4.6–14.2)	10.3 (5.1–14.7)	0.729
Blood-collection time after stroke (h)	11.8 (6.7–16.0)	11.6 (6.5–16.6)	0.975
Blood glucose levels (mmol/L)	12.7 (8.8–17.3)	10.3 (8.6–12.0)	< 0.001
Blood leukocyte count ( × 10^9^/L)	7.8 (6.5–10.4)	7.5 (5.9–11.3)	0.963
Serum HIF-1α levels > 384.0 pg/ml	70 (71.4%)	30 (18.9%)	< 0.001

Variables were reported as count (proportion), mean ± standard deviation, or median (percentiles 25th−75th) where appropriate. Statistical methods included the student t-test, Mann–Whitney test, Fisher's exact test, and χ^2^ test. HIF-1α means hypoxia-inducible factor 1alpha. Poor outcome was defined as extended Glasgow outcome scale scores of 1–4.

**Figure 8:**
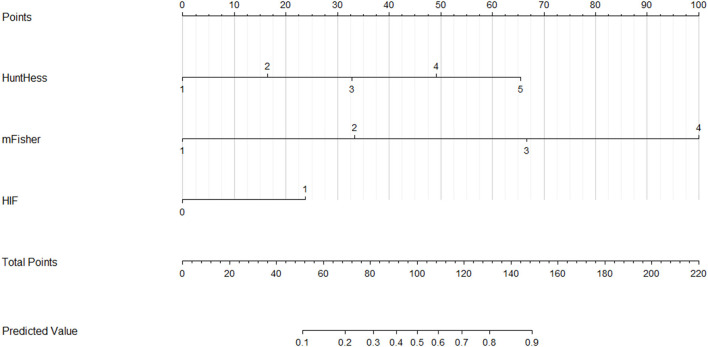
Nomogram for predicting poor outcome after aneurysmal subarachnoid hemorrhage. Serum hypoxia-inducible factor 1alpha levels combined with Hunt–Hess scores and modified Fisher scores significantly differentiated poor outcomes. For HIF, “1” indicates serum hypoxia-inducible factor 1alpha levels >384.0 pg/ml and “0” means serum hypoxia-inducible factor 1alpha levels < 384.0 pg/ml. HuntHess, Hunt–Hess scores; mFisher, modified Fisher scores; HIF, hypoxia-inducible factor 1alpha.

**Figure 9:**
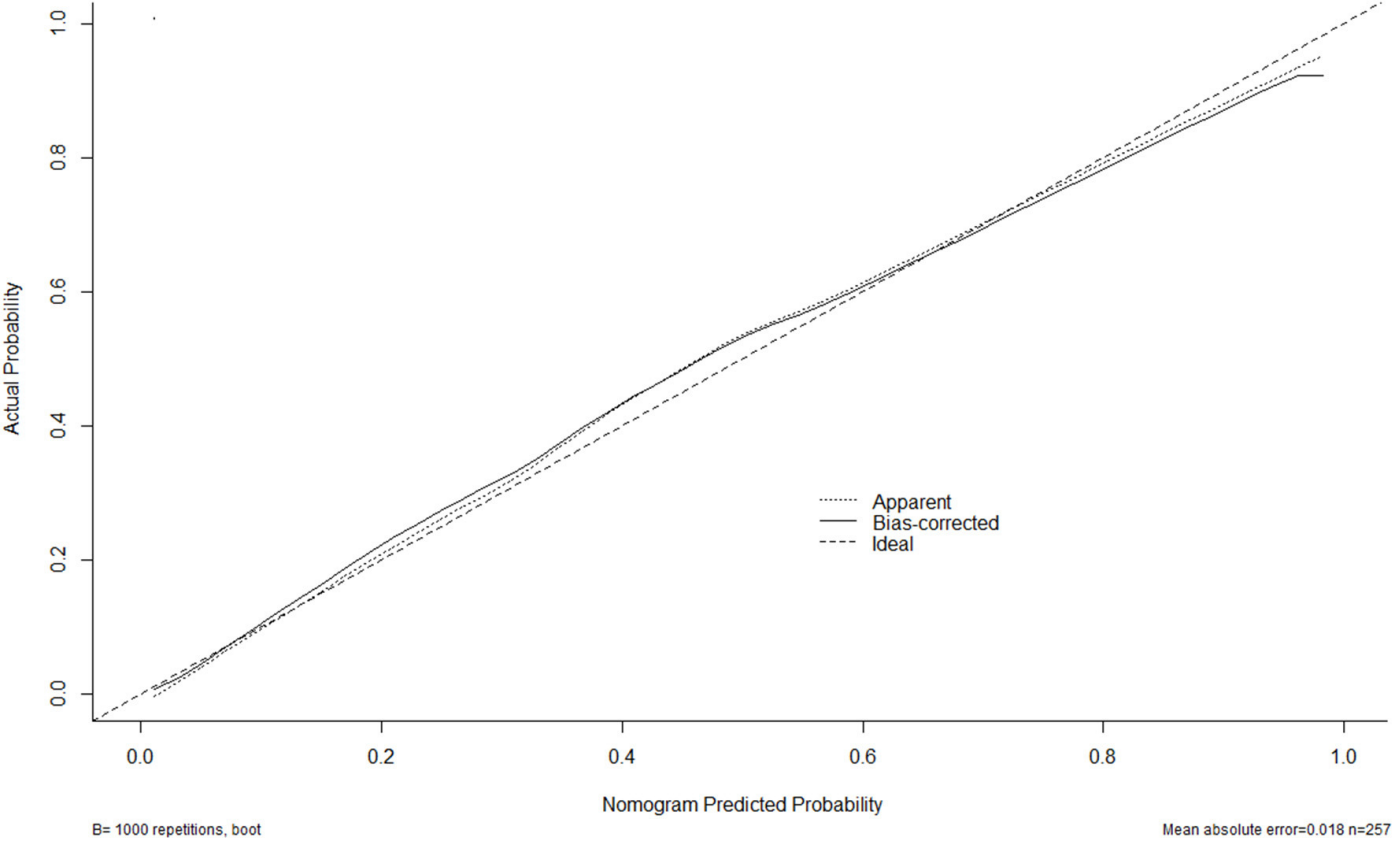
Calibration curves of the nomogram for predicting poor outcome after aneurysmal subarachnoid hemorrhage. The *x*-axis denotes the predicted poor outcome. The *y*-axis denotes the actual poor outcome. The diagonal dotted line means a perfect prediction using an ideal model. The solid line represents the performance of the nomogram, of which the closer fit to the diagonal dotted line indicates the better prediction of the nomogram.

**Figure 10:**
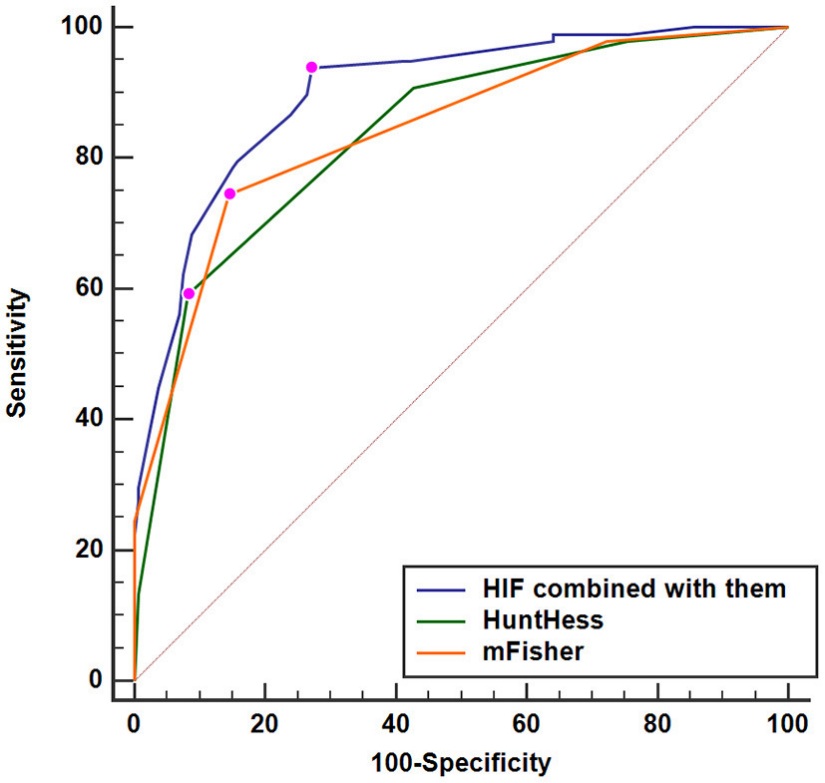
Predictive ability with respect to serum hypoxia-inducible factor 1alpha levels combined with Hunt–Hess scores and modified Fisher scores for poor outcome. Serum HIF-1α levels combined with Hunt–Hess scores and modified Fisher scores displayed substantially elevated discriminatory capability for poor outcome in comparison to Hunt–Hess scores and modified Fisher scores alone (*P* < 0.01). HuntHess, Hunt–Hess scores; mFisher, modified Fisher scores; HIF, hypoxia-inducible factor 1alpha.

A nomogram was plotted to get a more comprehensive view of the relationship between a poor prognosis and other independent predictors, namely, Hunt–Hess scores, modified Fisher scores, and serum HIF-1α levels of >384.0 pg/ml (Figure 8). The calibration curve of the nomogram for predicting a poor prognosis showed that the mean absolute error was 0.018 (Figure 9). The combined binary logistic regression model was built, which included Hunt–Hess scores, modified Fisher scores, and serum HIF-1α levels of >384.0 pg/ml (using Hosmer and Lemeshow test, *P* = 0.604). Subsequently, serum HIF-1α levels of >384.0 pg/ml combined with Hunt–Hess scores and modified Fisher scores had AUC at 0.900 (95% CI: 0.862–0.938). In Figure 10, the prognostic predictive ability of their combination was significantly superior to those of Hunt–Hess scores (AUC, 0.839; 95% CI: 0.791–0.886; *P* < 0.001) and modified Fisher scores (AUC, 0.844; 95% CI: 0.799–0.890; *P* < 0.001).

## Discussion

To the best of our knowledge, this is the first series for investigating circulating HIF-1α levels in humans with aSAH. Subsequently, we found some intriguing results that (1) there was a substantial increase of serum HIF-1α levels after aSAH, which were independently correlated with illness severity indicated by Hunt–Hess scores and modified Fisher scores, (2) serum HIF-1α levels independently predicted DCI and 6-month poor outcome after aSAH, and (3) serum HIF-1α levels combined with Hunt–Hess scores and modified Fisher scores displayed significantly higher predictive ability for DCI and poor 6-month outcome than Hunt–Hess scores and modified Fisher scores alone under the ROC curve. Such data were strongly supportive of the notion that serum HIF-1α may serve as a useful prognostic biomarker of aSAH and can aid in severity assessment and prognostic prediction after a human with aSAH.

Hypoxia-inducible factor 1alpha is a crucial upstream transcriptional factor in response to hypoxia (10). HIF-1α may act as a double-edged sword to participate in pathophysiological processes after acute brain injury (15–21). Some experimental data have demonstrated that HIF-1α may confer brain detrimental effects *via* increasing neuronal apoptosis and disrupting blood–brain barrier permeability (15–17). Contrarily, other experimental studies have shown that HIF-1α may harbor brain-protective potential and the protective mechanisms may be related to HIF-1α-induced angiogenesis and glycolytic metabolism (18–21). HIF-1α expression by rat-cultured cortical neurons was upregulated after being deprived of oxygen and glucose (27). Also, HIF-1α messenger RNA was induced in the ischemic brain penumbra of rats with permanent middle cerebral artery occlusion (28). Similarly, the HIF-1α protein was obviously expressed in perihematomal neurons of rats with intracerebral hemorrhage (14). Moreover, HIF-1α was expressed predominantly in neurons of rats subjected to endovascular perforation to cause aSAH (15). Overall, HIF-1α may be mainly derived from neurons after acute brain injury. Because HIF-1α, functioning as a transcription factor, is localized in the intracellular compartment (12, 13), hypothetically, in response to acute brain injury, HIF-1α in the extracellular compartment may not be secreted from neurons but be released from destroyed neurons. We found a substantial elevation of serum HIF-1α levels after aSAH. It is deduced that HIF-1α in peripheral blood of this group of patients with aSAH might at least partly originate from hemorrhage-injured neurons.

The neurovascular unit is composed of various interacting cells, including neurons, astrocytes, microglia, pericytes, endothelial cells, and vascular smooth muscle cells (29). Neurovascular unit components interact and balance microenvironments, thereby ensuring stable neuronal function (30). HIF-1α is extensively expressed in the neurovascular unit and mediates the transcriptional expression of more than 100 genes under hypoxic conditions (31). These HIF-1a target genes are implicated in various processes, including inflammation, cell metabolism, proliferation, survival, death, cytoskeletal structure formation, cell adhesion, and movement (32). Thus, HIF-1α exerts cell type-specific actions and its phenotype becomes more complex. Specifically, HIF-1α harbored neuroprotective potentials *via* protecting cerebrovascular function, lessening cerebral edema, and inhibiting neuronal apoptosis after experimental aSAH (33–35); in contrast, HIF-1α conferred detrimental properties *via* disrupting the blood–brain barrier, increasing brain edema, and aggravating neuroinflammation in animals with aSAH (36–39). Overall, such divergent effects of HIF-1α in aSAH may be attributed to the diversity of downstream targets, which function in different cell types within the neurovascular unit. Also, such differences in the functioning of HIF-1α between cell types and in different environments make it a challenging therapeutic target in aSAH.

Up to now, there have been three clinical studies regarding the relationship between serum HIF-1α levels and the prognosis of humans with acute brain injury, where elevated serum HIF-1α levels were highly correlated with cerebral infarction size in 40 patients with acute ischemic stroke (22), were independently associated with admission Glasgow outcome scale scores and 90-day poor outcome in 97 patients with acute spontaneous intracerebral hemorrhage (23), and were independently predictive of 90-day poor prognosis in 104 patients with severe traumatic brain injury (24). In the current study of patients with aSAH, a multicenter study was conducted, the sample number was increased to 257 patients, the follow-up time was extended to 6 months after injury and its associations with both severity and prognosis were verified using multivariate analyses, and the combined model was built for prognostic prediction. Our data demonstrated that elevated serum HIF-1α levels, in strong correlation with Hunt–Hess scores and modified Fisher scores, were independently associated with DCI risk and long-term poor clinical outcomes. Of note, a combined model, containing serum HIF-1α levels, Hunt–Hess scores, and modified Fisher scores, displayed higher predictive ability for DCI development or poor functional outcome following aSAH in comparison to Hunt–Hess scores and modified Fisher scores alone. In summary, serum HIF-1α may represent a potential prognostic biochemical marker of aSAH.

There are several limitations of this study. First, although our data were demonstrated in a multicenter study, which included a medium sample size of patients with aSAH, a larger cohort study is needed to validate the conclusions. Second, except for intraventricular hemorrhage and hydrocephalus, some other acute adverse events, such as epilepsy, hyponatremia, and pulmonary infection, have not been recorded in this study. Thus, a future study is warranted, which will contain more acute adverse affairs. Finally, serum HIF-1α levels were determined at hospital admission of patients with aSAH. Thus, its dynamic changes in serum levels are unclear, which will be explored in future.

## Conclusion

To the best of our knowledge, this is the first series for investigating circulating HIF-1α levels in humans with aSAH. In this multicenter study, we demonstrate that elevated serum HIF-1α levels are strongly correlated with illness severity and are tightly associated with DCI and long-term functional outcomes of aSAH. Interestingly, serum HIF-1α levels combined with Hunt–Hess scores and modified Fisher scores have a higher predictive ability for DCI risk or poor functional outcome after aSAH, as compared to Hunt–Hess scores and modified Fisher scores alone. Hence, serum HIF-1α may display the prognostic role and be of clinical value in severity assessment and outcome prediction of aSAH.

## Data availability statement

The raw data supporting the conclusions of this article will be made available by the authors, without undue reservation.

## Ethics statement

The studies involving human participants were reviewed and approved by the Ruian People's Hospital, Ningbo Branch, Ren Ji Hospital, Shanghai Jiao Tong University School of Medicine, and Hangzhou Ninth People Hospital. The patients/participants provided their written informed consent to participate in this study.

## Author contributions

All authors listed have made a substantial, direct, and intellectual contribution to the work and approved it for publication.
